# Filling the gaps in global antimicrobial resistance research/surveillance

**DOI:** 10.1186/s12879-019-4708-6

**Published:** 2020-01-14

**Authors:** Luis Furuya-Kanamori, Laith Yakob

**Affiliations:** 1 Research School of Population Health, Australia National University, Canberra, ACT Australia; 2 Faculty of Infectious & Tropical Diseases, London School of Hygiene & Tropical Medicine, London, UK

There is a large clinical and public health burden associated with antimicrobial resistance (AMR). This burden is likely to increase over time and urgent action is required [1, 2]. In a recent review, it was estimated that AMR could cause 10 million deaths a year by 2050 [3]. However, there are major uncertainties associated with this estimate [4], not least of all the patchiness of surveillance data especially when it comes to developing countries.

A collection of new articles has been brought together in this special issue for *BMC Infectious Diseases*. This journal being an obvious hub for this type of research: being open access broadens the audience; and the board of committed, expert editors lends rigour to the science that is communicated. Emphasis has been placed on presenting latest results pertaining to AMR from around the world. Going to press, this special issue already has articles from a dozen countries spanning Latin America, Europe, West-to-East Africa, the Middle East, South and East Asia.

The criticality of surveillance in AMR is widely acknowledged and all of the articles of the special issue fall under this umbrella term, across very different scales, ranging from Irenge et al. [5] reporting on multidrug-resistant (MDR) *E. coli* in local healthcare facilities in South Kivu province of the Democratic Republic of Congo to Galani et al. [6] reporting nationwide hospital carbapenem resistant *K. pneumoniae* survey for Greece.

Comprising one of the five strategic priorities of the Global Action Plan (GAP) on AMR [7], a recent report from the Interagency Coordination Group on Antimicrobial Resistance [8] describes several ways in which surveillance can support efforts to reduce AMR:

i) **detect the emergence and prevalence of AMR** e.g. Nelson et al. [9] developed a rapid cost-effective molecular detection test for macrolide resistance that can be implemented in clinical settings. Surveillance studies at a national [6], regional [10], and local [5] level have exposed the magnitude of the AMR problem around the world.
ii) **guide patient treatment** e.g. Arevalo-Jaimes et al. [11] found 38% of Colombian patients with *H. pylori* were resistant to first line drug clarithromycin, calling into question the appropriateness of the current, standard triple therapy. Likewise, Mashe et al. [12] found that 88% of *S. typhi* in Zimbabwe were resistant to two or more first line drugs indicating the need for changes in their current guidelines.
iii) **identify populations at risk** e.g. Milovanovic et al. [13] found that more than half of patients with liver cirrhosis in Serbian tertiary care facilities that contracted hospital-acquired urinary tract infections were infected with MDR strains, calling for individualized protocols for treatment of these immunocompromised populations.
iv) **inform policy development** e.g. Haddad et al. [14] from the Lebanese Society of Infectious Diseases and Clinical Microbiology publish new guidelines for empiric and targeted antimicrobial therapy of complicated intra-abdominal infections based on risk factors, site of acquisition of infection, and clinical severity of illness.
v) **assess the impact of interventions.** Although there were many studies that investigated the current state of AMR to plan interventions, none described intervention effectiveness.

This being a rolling special issue, our coverage is expected to expand even further and continue spotlighting findings from regions of the world for which data are largely absent, and yet most necessary according to burden projections [3]. Given its conspicuous absence both from this special issue as well as the wider literature, we particularly encourage the submission of articles that assess AMR intervention effectiveness.
